# Performance in the accuracy task in children with Childhood Apraxia of Speech after an integrated intervention of literacy and motor skills

**DOI:** 10.1590/2317-1782/20212021126

**Published:** 2022-01-05

**Authors:** Karina Carlesso Pagliarin, Marileda Barichello Gubiani, Rafaela Rossini Rosa, Márcia Keske-Soares

**Affiliations:** 1 Universidade Federal de Santa Maria – UFSM - Santa Maria (RS), Brasil.

**Keywords:** Apraxia, Speech Therapy, Children, Literacy, Motor Skills, Apraxia, Fonoaudiologia, Crianças, Alfabetização, Habilidades Motoras

## Abstract

Difficult in literacy skills are often seen in children with Childhood Apraxia of Speech (CAS). This occurs because oral language has direct relationship with reading and writing learning. The purpose of this study was to evaluate the performance in the accuracy task of an integrated phonoarticulatory awareness, motor skills and literacy intervention of three children with CAS. Three boys between 5;3 and 5;8 years of age, with CAS, were offered 2 hours per week of therapy sessions based on literacy and motor skills. The children were assessed before and after therapy and at a maintenance assessment 1 month after the treatment ceased. The children improved on the accuracy task considering their deficits level. Improvement was maintained at the maintenance assessment. Therapy based on literacy considering phonoarticulatory awareness and motor skills can help children with CAS, but the severity of the children’s communication problems must be taken into consideration.

## RESUMO

Dificuldade nas habilidades de alfabetização são frequentemente observadas em crianças com Apraxia da Fala na Infância (AFI). Isso ocorre porque a linguagem oral tem relação direta com a aprendizagem da leitura e da escrita. O objetivo deste estudo foi avaliar o desempenho na tarefa de acurácia de uma intervenção integrada de consciência fonoarticulatória, habilidades motoras e letramento de três crianças com AFI. Três meninos com idade entre 5:3 e 5:8 anos, com AFI, receberam 2 horas semanais de sessões de terapia baseadas na alfabetização e habilidades motoras. As crianças foram avaliadas antes e após a terapia e em uma avaliação de manutenção 1 mês após o término do tratamento. As crianças melhoraram na tarefa de precisão, considerando o nível de seus déficits. A melhora foi mantida na avaliação de manutenção. A terapia baseada na alfabetização, considerando a consciência fonológica e as habilidades motoras, pode ajudar as crianças com AFI, mas a gravidade dos problemas de comunicação das crianças deve ser levada em consideração.

## INTRODUCTION

It is known that Childhood Apraxia of Speech (CAS) is a failure in the programming and planning of speech movements. In addition, this disorder can result in speech and language difficulties ^(1)^. Children with CAS are at risk for language, reading and writing difficulty ^(2)^. They have poorer phonoarticulatory awareness skills than children without this disorder ^(3)^.

There are different types of approaches that can be used in the treatment of CAS, as motor learning techniques, linguistic or multimodal (such as augmentative/alternative communication methods). Regardless of the approach chosen, the method should favor the child's best skills ^(2)^.

Based on this, many studies have shown that intervention should not be limited to speech motor difficulties. Therefore, techniques that supply phonological awareness, rather than imitation strategies, may be more appropriate and effective in children with CAS ^(2,4)^. CAS intervention studies are important because no specific treatment has been found to be effectively superior ^(2)^. Generally, the treatments are based on linguistic and/or motor skills ^(1)^.

In a review ^(2)^, many types of motor-based methods were found: The Dynamic Temporal and Tactile Cueing treatment method, the PROMPT system, the Melodic Intonation Therapy, the Nuffield Dyspraxia, the Rapid Syllable Transition Treatment, traditional articulation-based drill therapy, rate control therapy, adapted cueing technique, and integral stimulation or dynamic temporal and tactile cueing.

Linguistic therapy generally emphasizes awareness of the phonological components of a word in addition to motor repetition ^(3)^. Children with CAS can have a deficit in phonological memory, as well as in reading and writing decoding skills ^(3)^.

However, few studies seek to prevent learning difficulties at earlier stage, that is, before the child’s entry into school, especially children with CAS.

The negative effects of early reading difficulties are related to unsolved speech and language impairments, combined with poor phonoarticulatory awareness skills. Such difficulties place these children at significant risk for persistent reading difficulties in later school years ^(5)^. Therefore, the purpose of this study was to evaluate the performance in the accuracy task of an integrated phonoarticulatory awareness, motor skills and literacy intervention of three children with CAS.

## CASE PRESENTATION

Three male children participated in this study (Oscar, Chris, and Philip; pseudonyms). They are Brazilian Portuguese speakers, between 5:3 and 5:8 years old at first evaluation. In Brazil, children typically begin formal education at age of 6 years old. In this situation, the participants had no received classroom instruction before entering the study. All children and their parents have signed the Informed Consent Form, under research project protocol n. 16239413.0.0000.5346, agreeing with the completion and dissemination of the study and its results.

Children were initially assessed by a Speech-Language Therapist (SLP) when they were 3:0 years old initially diagnosed with specific language impairment. Speech-Language intervention was recommended, and they were attended together (group intervention) for two years, once a week. Their parents were then advised that further therapy was necessary, as they had made little progress. Thus, they were transferred to Speech-Language and Hearing Clinic at University in 2015.

### Assessment

The first and second authors assessed children when they were five years old. Evaluations were done in one-hour sessions over three sessions in a quiet room at clinic. All then met the criteria to be eligible participants for the study, which was: no structural problems in the speech organs; no signs of dysarthric symptoms; and no vision or hearing problems. The hearing of participants was screened using a portable pure tone audiometer INTERACOUSTICS - AD 229 administered at 24 decibels hearing threshold level for the frequencies of 500, 1000, 2000, and 4000 Hertz.

First, a complete interview was conducted with the parents, to verify important data on pregnancy, childbirth, neuropsychomotor development and language acquisition, as well as interaction with the child, through free play.

Furthermore, participants were assessed by *INFONO: Phonological Assessment Instrument* (Instrumento de Avaliação Fonológica), which is a computerized test ^(5)^ that consists of 80 animated figures, individually presented to child naming, to determine the sound substitutions and phonemic inventory. The motor productions abilities were assessed using Dynamic Evaluation of Motor Speech Skills - Brazilian version (DEMSS-BR) ^(6)^. The phonoarticulatory awareness was assessed using a standardize protocol – *The Articulatory Awareness Instrument* (Instrumento de Avaliação da Consciência Fonoarticulatória – CONFIART) ^(7)^. Phological discrimination was assessed using a standardize protocol – *Phological Discrimination Test* (Teste de Discriminação Fonológica) ^(8)^. The receptive and expressive vocabulary was assessed using *Receptive Vocabulary Test* (Teste de Vocabulário Auditivo - TVAud 33o) ^(9)^ and *Naming Test for Children* (Teste Infantil de Nomeação) ^(10)^.

The diagnose of CAS was made by the first author through the DEMSS-BR. The first author reviewed all audio files (collected by the second author) and checked all scores. Mean inter-rater agreement was 90%. After one month without intervention all children were re-evaluated on DEMSS-BR to confirm or not the acquisitions maintenance. Table 1 shows descriptive data of children.

**Table 1:** Summary of participants’ characteristics

	**Oscar**	**Chris**	**Philip**
Age	5;3	5;3	5;8
Gender	Male	Male	Male
Relevant information	Postnatal anoxia	Severe anemia	Idiopathic CAS
Phonoarticulatory awareness –CONFIART (/16)	03 (deficit)	07 (deficit)	08 (average-low)
Phonemic Discrimination (/23)	21 (average)	22 (average)	21 (average)
Naming test (/60)	06 (deficit)	20 (average)	26 (average)
Receptive Vocabulary (/33)	28 (deficit)	32 (average)	30 (average)
Phonemic inventory	Initial: t, g, m, n Medial: b, ʃ, ʒ Final: N, L	Initial: p, d, k, z Medial: p, t, d, s, ʃ, n Final: L, s	Initial: p, b, t, d, k, g, f, v, s, z, ʃ, ʒ, m, n, R, L Medial: p, b, t, d, k, g, f, v, s, z, ʃ, ʒ, m, n, L Final: N, L, s, r

The results of standardized tests and speech characteristics for each participant are presented in Table 1 and 2. Tables 1 and 2 demonstrate that the three children had varying linguistic profiles, consistent with the syndromic nature of CAS ^(1)^.

**Table 2:** DEMSS-BR scores

**Accuracy (Total)**	**Oscar**	**Chris**	**Philip**
Vowel-Vowel (/20)	16	20	20
Consonant-Vowel (/40)	23	31	33
Reduplicated Syllable (/16)	16	12	16
Consonant-Vowel-Consonant (/16)	3	12	12
Bisyllabic – 1 Consonant-2 Vowels (/20)	6	16	20
Bisyllabic more varied syllabic shape (/32)	20	23	32
Multisyllabic (/32)	5	12	26
Total (/176)	89	126	159

### Intervention

The first author instructed three SLP undergraduate students in clinical supervision to the implementation of the intervention for the participants. SpeechLanguage therapy started 15 days after the assessments. The children received fifteen sessions of the research intervention in individual sessions over two months. The sessions took place twice a week and lasted approximately 50 minutes. The first author observed the sessions and helped if there was any difficulty on the part of the therapist. To ensure treatment fidelity, SLP were required to fill out a session completion worksheet after each session.

The intervention had three aims: (1) reduce speech error patterns at the single word level and in connected speech; (2) improve phonoarticulatory awareness; and (3) development literacy skills.

### Structure of the sessions

The therapy was based on *Scliar Literacy Method* (Método Scliar de Alfabetização). One of its principles is that letters should not be taught in isolation and by name; but by the phoneme that one or two graphemes represent and inside the word ^(11)^. Learning follows an order of increasing complexity and according to the method starting with the graphemes V, I, O. These three graphemes are symmetric and easier to learn ^(11)^. Furthermore, all three graphemes represent phonemes which sounds may be produced continuously.

In each session, only one phoneme was presented and, to reinforce it, visual cues (visual association letter with figures, letter of different colors), tactile (different textures, such as clay, sandpaper) and earphone were used. The phoneme was presented in isolation and at different frequencies so that the child could understand it properly and try to reproduce. The sound was associated with the articulatory gestures and movements during their production. Visual, auditory and tactile cues related to hand movements and speech are use in the introduction of a new grapheme, since the children were not previously literate.

The child had a small mirror at his disposal that allows him to see only his mouth. Besides, the children had a figure of a mouth with the point of articulation of the sound worked. They were instigated by the SLP to perform the correct production of the target sound, initially in isolation, passing to the word and finally to reading.

After graphemes and their sound values were taught, graphemes were taught in isolated words which were part of a story built during the book *Vivi’s Adventures* (Aventuras de Vivi) ^(12)^. At that moment, the child conducts training in reading and articulatory production of an isolated word and then the SLP reads the story to the client. There were words used in it that the client had already learned. Subsequently, the retelling of the history read by the therapist was requested, as a way of subjectively evaluating the interpretation of the text and, consequently, spontaneous speech.

As children progress, more words are bolded until the entire text is highlighted and the child can read the text alone. Whenever the child was able to read the target word correctly, he could make associations with other words in his vocabulary that were phonetically similar. Although there is only one phoneme and its graphical representation is presented per session, the phonemes and graphemes already learned were taken up, to assist in reading by lexical route. The reading of the words, in addition to the production of the articulatory gesture, also sought to break co-articulation, that is, the child was induced to read the word with more prosody, without syllabizing.

In addition to promoting awareness of sound and reading of words, the sessions also addressed a variety of cues, teaching sound by sound, breaking words into syllables and using cued articulation. If a speech production error occurred in the activities, the SLP showed again the correct production. Data was analyzed descriptively.

Table 3 shows the total number of therapy sessions for each boy and the total number of sounds targeted in those sessions.

**Table 3:** Therapy sessions summary

	Oscar	Chris	Philip
Number of sounds worked	6	6	16
Total Number of therapy sessions	15	15	15
Number of book units worked (/24)	3	3	9

Oscar and Chris had more difficulties in this kind of therapy approach probably because they have more severe degrees of CAS as shown in DEMMS-BR performance. However, Philip had a great progress during the sessions. In each session new sounds were worked with him, which means that no target sound had to be repeated (Table 3).

Table 4 shows the performance of three children in DEMSS-BR protocol pre- and post-therapy and maintenance assessment.

**Table 4:** Comparison of baseline, post-therapy, and maintenance assessment on DEMSS-BR

	**Baseline**				**Post-therapy**				**Maintenance**		
**DEMSS-BR Accuracy (Total)**	**Oscar**	**Chris**	**Philip**	**Oscar**		**Chris**	**Philip**	**Oscar**		**Chris**	**Philip**
Vowel-Vowel (/20)	16	20	20	20		20	20	20		20	20
Consonant-Vowel (/40)	23	33	33	32		34	39	34		34	40
Reduplicated Syllable (/16)	16	12	16	16		13	14	16		14	16
Consonant-Vowel-Consonant (/16)	8	12	12	7		13	14	16		14	16
Bisyllabic – 1 Consonant-2 Vowels (/20)	6	16	20	8		17	20	4		16	20
Bisyllabic more varied syllabic shape (/32)	20	23	32	14		20	30	17		28	32
Multisyllabic (/32)	5	12	26	5		20	32	7		22	32
Total (/176)	89	126	159	102		137	169	114		148	176

For each boy speech sound errors decreased, and sequencing abilities increased significantly after the sessions. Besides, improvement was maintained after one month.

## DISCUSSION

The purpose of this study was to evaluate to evaluate the performance in the accuracy task of an integrated phonoarticulatory awareness, motor skills and literacy intervention of three children with CAS. The intervention aimed to develop phoneme awareness, increase knowledge of phoneme grapheme relationships, and improve speech. Outcome was positive for all three children although the rate of progress varied.

Oscar’s intervention over eight weeks led to decrease in inconsistency from 87% to 74% and at maintenance evaluation inconsistency decreased was 62%. Chris’s over eight weeks led to decrease in inconsistency from 50% to 39% and at maintenance evaluation inconsistency decreased was 28%. Philip’s intervention over eight weeks, led to decrease in inconsistency from 17% to 7% and at maintenance evaluation inconsistency totally decreased (0%). These findings suggest that this kind of therapy approach was effective for the remediation of inconsistence speech sounds. However, children with more severe CAS like Oscar and Chris had more difficulties with phonoarticulatory awareness when associate to reading skills, they did not progress on book tasks as Philip. Children with CAS have been found to display phonological awareness deficits at the syllable and phoneme level on both receptive and expressive tasks ^(2)^. This contributes for their reading and spelling learning delay/deficits ^(3)^.

Something interesting in this treatment is that the phonemes are taught with meaning within words, like a previous study ^(13)^. However, this therapy adds reading as another resource for children to learn, besides the correct articulation movement.

Skelton and Hagopian ^(13)^ used fricatives (f, v, s, z) in a randomized treatment of children with CAS showing favorable results. Although the current study used another form of treatment, the fricative /v/ was the first one emphasized being effective because its articulation and perceptual characteristics ^(2)^. However, Oscar and Chris needed more sessions to establish target sound in a single word, while Phillip took one session, he had the most rapid gains.

Children with CAS may have severe and persistent phonoarticulatory awareness and phonological processing difficulties ^(3)^. Thus, they can respond positively to phonoarticulatory awareness intervention taking into consideration that phonoarticulatory awareness is especially important to reading and spelling acquisition ^(4)^.

As showed in Table 1 Oscar’s presented deficit in receptive vocabulary test, this seems to have influenced his results in intervention program. This result is like McNeill et al. ^(14)^ findings in which two children with receptive vocabulary standard scores below the typical range made the least progress in their speech development over the intervention. Oscar’s language and motor oral skills were more severely impacted than the other two participants. Consequently, intervention may have been an inadequate period for acquisition of new phonemes considering the severity of his apraxia.

Some limitations need to be noted and addressed in future research. The results from this study are from a limited sample of participants and was non-controlled. Another limitation is to have established only 15 sessions for each child. We know that is not enough for CAS, maybe for mild disorder it can be appropriate as it was for Philip, but not for severe disorders. It requires more time.

The current findings have important clinical implications for SLP working with children with CAS. The findings show that it is possible to simultaneously stimulate speech production, phonoarticulatory awareness, and literacy skills in this population. The data also indicated that therapy sessions based on literacy and motor skills is an efficient resource for participants with CAS. However, these results should be carefully analyzed considering the effects of this intervention in just three participants. Furthermore, future research should focus on establishing the efficacy of thi approach through well-designed group experimental study that provide adequate descriptions of participants and control for the influence of variables outside of treatment.

## FINAL COMMENTS

The case studies presented on this study focus on children with CAS who appear to have a deficit in phonoarticulatory awareness. A literacy and motor speech approaches to intervention that focuses on awareness of sound and reading of words were shown to remediate the speech of three children considering accuracy and consistency. Although it is important to note that there are differences among children reported here since Philip had mild deficits in comparison to others. However, the approach was shown positive results for all three children considering their difficulties.
